# The effects of interactions between intrinsic properties and network parameters on bilateral phasing in a reduced leech heartbeat system

**DOI:** 10.1186/1471-2202-15-S1-P13

**Published:** 2014-07-21

**Authors:** Adam L Weaver

**Affiliations:** 1 Department of Biology, Saint Michael's College, Colchester, VT 05439, USA

## 

The leech heartbeat central pattern generator (CPG) consists of a network of heart interneurons (HN) that coordinate heart excitor (HE) motor neuron activity via inhibitory chemical synapses. Each segmental pair of HE’s is connected to one another via electrical coupling. Depending on the segment, the pair of motor neurons in the living system is active across a wide range of phase differences from nearly in-phase to anti-phase [1]. Prior efforts to model this complete network have not quantitatively matched all contralateral phase values observed [2]. We have created a reduced network model in Simulink to explore parameters that contribute to these phase differences. This parameter search has been analyzed in MATLAB using the PANDORA toolbox [3].

**Figure 1:**
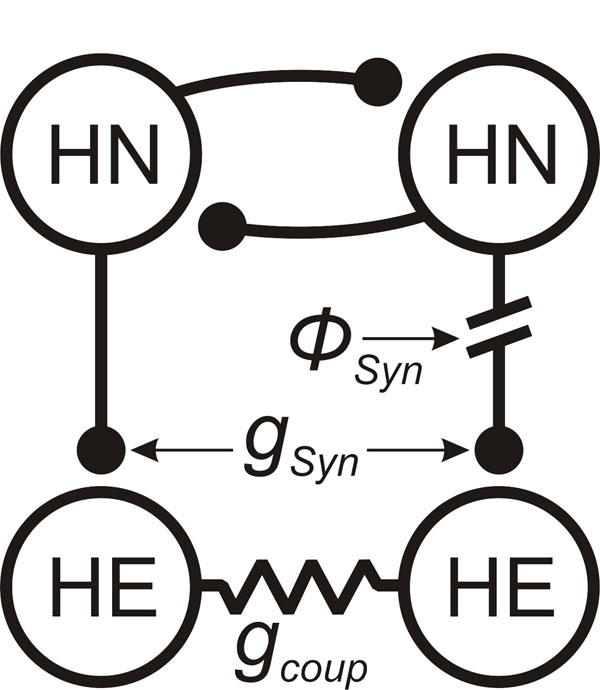
Leech heartbeat circuit diagram showing identified CPG heart interneurons (HN) and heart excitor motor neurons (HE).

In our network model, we implemented known neuronal properties and synaptic connections from a single segmental ganglion. The HN’s were modeled as endogenous bursters as previously described with a network cycle period of 9.45 ± 0.11 sec [4]; in previous studies [5], the HE’s were modeled as tonic firers. We varied three network parameters shown in Figure 1 in this study: phased delay of the right HN synaptic input [*Φ_Syn_*], the maximum conductances of the inhibitory synapse [*g_Syn_*], and electrical coupling strength [*g_coup_*]. We varied *Φ_Syn_* to emulate the variable timing of inhibition onto HE’s in the full network of the living system. In addition, we varied the set of maximum conductances for the HE neurons in a linear fashion from classical HE (tonic firing) to HN (endogenous bursting) values to ascertain the role of intrinsic properties on phase-shifts.

This linear variation of HE properties from tonic firing towards endogenous bursting led to a step-wise phase advance up to ~0.10 for both neurons. In-phase (*Φ_Syn_*: 0.0) and anti-phase (0.5) values of *Φ_Syn_* produced expected HE phase differences. However, intermediate synaptic delays (0.25, 0.75) caused a reduction in phase differences as the two motor neurons pull towards one another due to their electrical coupling. The amount of phase difference reduction was directly impacted in a nonlinear manner by the intrinsic properties of these HE neurons. Duty cycle (burst duration / cycle period) was not significantly impacted by intrinsic properties. In summary, increases in *Φ_Syn_* led to non-monotonic changes in HE motor neuron contralateral phase differences.

Our search of parameter space continues to provide a foundation for understanding the mechanisms underlying variable phase differences in neuronal networks and reinforces the importance of interactions between endogenous properties and synaptic connections for producing functional motor patterns. The project described was supported by Vermont Genetics Network through an NIGMS (NIH) Institutional Development Award P20 GM103449.
